# Exploring self-care and cervical cancer prevention attitudes and practices among Moroccan and Pakistani immigrant women in Catalonia, Spain: a comparative qualitative study

**DOI:** 10.1186/s12889-023-17445-2

**Published:** 2024-02-06

**Authors:** Jone G. Lurgain, Hakima Ouaarab-Essadek, Khadija Mellouki, Sumaira Malik-Hameed, Andleeb Sharif, Maria Brotons, Laia Bruni, Paula Peremiquel-Trillas

**Affiliations:** 1 Department of Public Health, Environments and Society, London School of Hygiene & Tropical Medicine, Keppel Street, WC1E 7HT, London, UK; 2 Cancer Epidemiology Research Programme, Catalan Institute of Oncology, Av Gran Via 199-203, 08908 L’Hospitalet de Llobregat, Barcelona, Spain; 3 Bellvitge Biomedical Research Institute – IDIBELL, Av Gran Via 199-203, 08908 L’Hospitalet de Llobregat, Barcelona, Spain; 4 Consortium for Biomedical Research in Epidemiology and Public Health – CIBERESP, Carlos III Institute of Health, Av. De Monforte de Lemos 5, 28029 Madrid, Spain; 5 Faculty of Medicine, University of Barcelona, C/ Casanova, 143, 08036 Barcelona, Spain; 6 Community & Public Health Team (ESPIC), Drassanes-Vall d’Hebron Centre for International Health and Infectious Diseases, Carrer de Sant Oleguer, 17, 08001 Barcelona, Spain

**Keywords:** Self-care, Prevention, Cervical cancer, Screening, Migrant health

## Abstract

**Background:**

Self-care and preventive health strategies may trigger health inequities when individuals’ cultural values and health beliefs are not fully understood and considered. In the case of cervical cancer (CC) screening programs immigrant women have shown lower attendance compared with native women, which increases the risk of late diagnosis and, consequently, a lower probability of survival. HPV self-sampling for CC screening has been recently added to the World Health Organization’s (WHO) list of self-care interventions as a promising tool to reduce this disparity and improve screening coverage. In Catalonia, Spain, the introduction of HPV self-sampling as a part of the new population-based CC screening program, is a significant step. However, there is a lack of research addressing self-care and prevention among immigrant populations in this region. This study aims to fill this gap exploring self-care and prevention attitudes and practices among Moroccan and Pakistani women.

**Methods:**

We conducted focus groups and individual interviews with 36 Moroccan and 37 Pakistani women in Barcelona, Spain. The topic guide of the focus groups included case vignettes to stimulate the discussion and a semi-structured questionnaire was used for the interviews.

**Results:**

Our findings show that most Moroccan and Pakistani women do not prioritize self-care and prevention. They seek care for symptom treatment rather than disease prevention. In this sense, they reported not having the habit of doing regular check-ups and their self-care and prevention attitudes and practices seemed to be conditioned by cultural values. The implementation of an effective call and recall system could enhance the engagement of these populations with CC screening services.

**Conclusion:**

This study provides evidence on how universal concepts of self-care and prevention may not aligned with more collectivist societies, emphasizing the limited applicability and motivation of global self-care interventions guidelines for individuals with different cultural backgrounds and values. Therefore, the successful implementation of CC screening programs or any other self-care intervention requires the adoption of culturally appropriate strategies.

## Background

Self-care and preventive health strategies empower individuals to take an active role in managing and assuming responsibility for their own health. However, this well-intentioned approach may inadvertently exacerbate health inequities when individuals’ cultural values, beliefs and health priorities are not well understood and enabling environments are lacking. Cervical cancer (CC) screening programs exemplify preventive care practices that inadvertently contributes to health disparities. Immigrant women have lower CC screening attendance compared with native women across different European countries, including Spain, increasing their risk of late diagnosis and treatment and, consequently, a lower probability of survival [39, 53]. Also, a higher prevalence of abnormal screening results among immigrant women compared to native counterparts, reveals the importance of prioritizing CC screening in these populations [52]. The World Health Organization (WHO) has recently added the HPV self-sampling method for CC screening to its list of self-care interventions as a promising tool to reduce this disparity and improve screening coverage [59, 60]. The impact of the COVID-19 pandemic on healthcare systems, exacerbating the existing challenges and constrains they face, and an increasing prevalence of chronic diseases worldwide have contributed to this new global self-care approach to preventive care, which traditionally relied on health professionals [7, 40, 43]. However, in increasingly multicultural societies it is crucial to adopt a more inclusive perspective to address self-care and preventive health concepts -often viewed from a ‘Western’ perspective- to implement effective health promotion interventions.

Self-care is a dynamic and multidimensional concept that has evolved over time [31, 41, 57]. For instance, different practices, such as cultural rituals, consumption of special foods to promote health and the intergenerational sharing of knowledge are examples of self-care strategies. However, it was not until the late twentieth century when the ability to provide care for oneself gained recognition in the global health arena and started to be applied to the management of chronic diseases [40]. Global health organisations use now a more holistic concept of self-care that encompasses individual and collective actions taken not only by people with an existing health problem to self-manage their illness, but also by healthy people to prevent disease and maintain health. The WHO latest definition of self-care is “*the ability of individuals, families and communities to promote health, prevent disease, maintain health and to cope with illness and disability with or without the support of a healthcare provider*” [58].

As the concept of self-care continues to evolve, numerous frameworks have emerged to guide the assessment of individuals’ engagement with self-care practices. For example, the International Self-Care Foundation [29] proposes seven domains of self-care: health literacy and health seeking behaviour; self-awareness of physical and mental health (e.g., engaging in health screening, monitoring blood pressure, etc.); adopting healthy eating; performing regular physical activity; risk avoidance (e.g., limiting alcohol consumption, getting vaccinated or practising safe sex); maintaining good hygiene; and ensuring a rational and responsible use of medicines.

The Catalan health system is aligned with the above WHO’s self-care and prevention approach and has targeted programs and resources on promoting a healthy diet, physical activity, mental health, vaccinations and screening [19, 20]. However, despite the availability of these services, very few studies in Catalonia, Spain, have addressed immigrant women’s perceptions, beliefs and practices about self-care and preventive care. These ‘Western’ models of self-care may not adequately apply to understandings of self-care among immigrants. Healthcare providers and policy makers must understand diverse cultural health beliefs in order to tailor health promotion interventions, including CC screening, to the needs of these groups and thus guarantee their access to quality preventive care. Therefore, the objectives of this study are to examine immigrant (Pakistani and Moroccan) women’s perceptions and beliefs related to health and illness, to explore their understanding of self-care and attitudes towards disease prevention, and to assess how these perceptions and health beliefs may influence self-care and prevention practices, especially CC screening.

## Methods

### Study design

We carried out a multi-method qualitative study [15, 16] combining focus group discussions (FGD) and semi-structured interviews (SSI) to explore Moroccan and Pakistani women’s health beliefs, self-care attitudes and prevention practices. This design was selected since the combination of research methods gave the participants the opportunity to choose their preferred mode of engagement in the research. For instance, the participants who did not feel comfortable taking part in a group conversation were given the option to undergo individual interviews. Moreover, this design facilitated the exploration of social dynamics and cultural understandings and values among participants regarding health-seeking behaviours through FGD [34]. At the same time, it enabled us to gather more nuanced and personal insights through SSI [23]. Additionally, we adopted a constructivist approach for this research, grounding our interpretations in the participants’ own views and experiences.

### Setting and study population

This study is embedded in a broaden CC screening implementation study in Catalonia, Spain, where the CC screening program is transitioning from opportunistic to population-based strategy and HPV self-sampling has been introduced as sampling method. Moroccan and Pakistani immigrant women represent the 3% (104,382) and 0.72% (17,416), respectively, of the total female immigrant population in Barcelona province [27, 28]. Both communities share the same religion and some social norms in their respective patriarchal societies. However, they have different cultural, linguistic and migration history -although in both groups many of them came to Catalonia through family reunification policies. Moroccan immigrants have a longer history of migration in Spain compared to Pakistani immigrants. The geographical closeness between Morocco and Spain and the international migration agreements between these two countries contributed to early waves of Moroccan immigrants -currently the largest immigrant community in Spain-, whereas Pakistan and Spain never had strong cultural and social bonds, nor economic agreements making the Pakistani migration history to Spain a more recent and distinct case [8, 35].

### Sample and participant recruitment

The study population consisted of 73 immigrant women born in Morocco and Pakistan, who migrated to Catalonia at 16 years old or older (thus not exposed to the public Spanish education system) and have lived in Catalonia for at least 1 year. Inclusion criteria for selecting the sample were the same as those used by the CC screening program in Catalonia: women aged 25 or that will turn 25 in the year of the study, to 65 who have never been diagnosed with this cancer. Participants were recruited in neighbourhoods of different municipalities: Raval (Barcelona city), La Mina (Sant Adrià del Besòs), Ca n’Anglada (Terrassa) and Torrassa (L’Hospitalet de Llobregat). All of them are socially deprived areas with high concentration of immigrants in the province of Barcelona [27, 28].

Convenience and purposive snowball sampling [21] were combined, based on the age, education background and length of stay in Spain. Recruitment was done through the networks of the community-based Research Assistants (RAs) from Morocco and Pakistan who were bilingual in Spanish or English and Urdu or Darija (participants’ native language) and were Psychology university students and members of the Community and Public Health team of the Drassanes - Vall d’Hebron Centre for International Health and Infectious Diseases, in Barcelona. RAs connected with religious and community associations and non-governmental organisations, as well as referring acquaintances. Recruitment continued until data saturation was achieved in both cohort of women (signifying that no additional data were found).

### Data collection

Data were collected between September and December 2022 through eight FGD and 22 SSI. Table 1 provides a detailed breakdown of the participation and languages used. FGD and SSI guides were informed by previous literature and adjusted to explore emerging topics and clarifying questions that respondents found difficult as data collection progressed. All FGD and SSI were audio-recorded and transcribed verbatim directly from Darija and Urdu into Spanish and English, respectively. The RAs as well as the rest of personnel implicated in the study were trained in ethical procedures and HPV and CC screening basic knowledge.

**Table 1:**
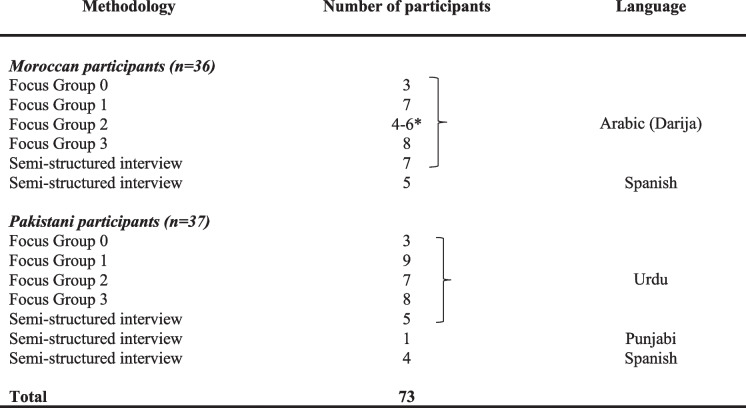
Breakdown of participation and languages used

*Two participants had to leave the focus group earlier as they had personal appointments.

All participants completed a self-administered questionnaire available in four languages (Spanish, English, Arabic and Urdu), capturing socio-demographic information, as well as Spanish language proficiency, religion identification, employment status and CC screening experience. A total of 24 Moroccan women and 27 Pakistani women participated in one of the eight FGD, which were moderated by two experienced community health providers (a nurse and a community health agent) who shared language and cultural background with participants. All FGD were conducted in Urdu or Darija, which were the native languages of Pakistani and Moroccan women, respectively. The FGD were organised by the community-based RAs through face-to-face contact and by phone and were facilitated in convenient and familiar places for the participants, such as community centres, faith-based associations and health facilities. Each group included between three and eight women and lasted around 2 hours. The topic guide included case vignettes to stimulate the discussion. Women were presented with the fictional case of Fatima, an immigrant woman diagnosed with CC, and were asked two questions related to her self-care and prevention attitudes and practices:

1 *Fatima was diagnosed with cervical cancer. What do you think she could have done to prevent cervical cancer?*

2 *Since her last son was born 8 years ago, Fatima did not visit the gynaecologist to do a check-up. Why do you think she did not go back to the gynaecologist and she did not undertake a Pap smear?*

The SSI (12 with Moroccan women and 10 with Pakistani women) were conducted by the RAs (KM and AS) and the first author (JGL). RAs were specifically trained in interview techniques and topic guides prior to the interviews. SSI length ranged from 35 to 75 minutes and were carried out in a place of convenience selected by participants (e.g., their homes, interviewers’ home, health facilities and religious centres). The SSI followed a semi-structured topic guide, which included topics such as participants’ self-perception of self-care, self-care and prevention attitudes and practices and motivations towards disease prevention.

### Data analysis

Thematic content analysis of each transcription was done using an inductive approach. The analysis process was initiated by conducting open coding on transcripts derived from two FGD and two SSI by the first author (JGL) and another investigator (PPT) independently. They read through each transcript, line by line, and made notes of broad themes and potential categories identified. Afterwards, the themes and sub-themes identified were discussed and they reached an agreement on an initial codebook. The first author coded the remaining transcripts using ATLAS.ti 23 software and developed a comprehensive final codebook with categories and subcategories. To ensure validation, the other investigator independently coded additional transcripts using the aforementioned final codebook. The analysis was organised based on themes and participants’ country of origin. Then the two sets of findings were compared. Table 2 provides a summary of the themes and sub-themes from the FGD and SSI. The transcripts from both the FGD and SSI were coded and analysed together, ensuring a comprehensive examination of the data. Socio-demographic information of study participants was described using STATA 16.

**Table 2:** Summary of the themes and sub-themes from FGDs and SSIs

Themes	Sub-themes
Theme 1:	
Understanding of health and illness	*• Health as the ‘absence of symptoms’* *• Health and illness is controlled by God’s will* *• Faith healing practices (*e.g.*, spiritual treatments or religious rituals)*
Theme 2:	
Self-care attitudes and practices	*• Prioritisation of family responsibilities* *• Lifestyle practices (*e.g.*, physical activity, healthy eating)* *• Religion as a self-care practice* *• Self-medication*
Theme 3:	
Knowledge and interest in disease prevention	*• Low awareness of disease prevention (*e.g.*, lack of interest/habit in performing regular check-ups)* *• Acceptance of early detection*
Theme 4:	
Motivators to engage with preventive care	*• Increasing awareness due to exposure to chronic diseases* *• Healthcare providers’ recommendation* *• Efficient call and recall system as facilitator*

### Ethical considerations and consent to participate

Prior to their participation, all respondents were provided with comprehensive information about the study and study procedures, and were informed about the confidentiality of the study. Each participant willingly to participate gave written informed consent. The study was approved by the Research Ethics Committees of the London School of Hygiene and Tropical Medicine (26186), Bellvitge University Hospital (PR 140/22) and Vall d’Hebron University Hospital (PR(AG)317/2022). To acknowledge their contribution and to facilitate transportation, participants were compensated with a public transportation 10-trip pass.

## Results

### Participants’ characteristics

Thirty-six women from the Moroccan community and 37 women from the Pakistani community participated in either FGD or SSI. Participants ages ranged from 24 to 65 years, with a median age of 40 years. Marital status varied among the participants, with 82.2% (*N* = 60) reported being married. Nearly all the Pakistani women (97.3%, *N* = 36) had children whilst among Moroccan participants, 77.8% (*N* = 28) had kids. Half of the participants had been living in Spain for at least 10 years (*N* = 36), while approximately 10% (*N* = 7) had arrived within the 2 years preceding the initiation of the study. The primary reason for migration to Spain among almost 85% (*N* = 62) was family reunification. Pakistani women had a higher level of studies than Moroccan women: 16 Pakistani participants had university studies, while only four Moroccan women had. Nine participants had no education (7 from Morocco and 2 from Pakistan). Regarding employment, a significant majority of the women, 80,8% (*N* = 59) were housewives and only 14 (12 from Morocco) were employed in either formal or informal jobs. In terms of Spanish language skills, approximately 37% of women (47.2% Moroccan and 27% Pakistani) reported not needing a translator during medical visits. Regarding religion, 90.4% of women considered themselves very or somehow religious and 97.3% identified themselves as Muslim. Up to 72.6% of the participants reported having been screened for CC at least once in their lifetime. Detailed information regarding participants’ characteristics can be found in Table 3.

**Table 3:** Socio-demographic characteristics of study participants and by country of origin (*n* = 73)

	Total participants		Morocco		Pakistan	
	N	*(%)*^a^	N	*(%)*^a^	N	*(%)*^a^
**Participants**^b^	73	*(100%)*	36	*(49.3%)*	37	*(50.7%)*
**Age. median (IQR)**^c^	42	*(35-48)*	39	*(33-46)*	40	*(34.5-47.5)*
**Age groups**						
24-34 years	18	*(24.7%)*	7	*(19.4%)*	11	*(29.7%)*
35-44 years	28	*(38.4%)*	13	*(36.1%)*	15	*(40.5%)*
45-54 years	19	*(26.0%)*	10	*(27.8%)*	9	*(24.3%)*
55-65 years	8	*(11.0%)*	6	*(16.7%)*	2	*(5.4%)*
**Level of studies**						
No studies	9	*(12.3%)*	7	*(19.4%)*	2	*(5.4%)*
Primary school	15	*(20.5%)*	9	*(25.0%)*	6	*(16.2%)*
Secondary school	25	*(34.2%)*	14	*(38.9%)*	11	*(29.7%)*
Vocational training	3	*(4.1%)*	2	*(5.6%)*	1	*(2.7%)*
University	21	*(28.8%)*	4	*(11.1%)*	17	*(45.9%)*
**Employment**						
Formal employment^d^	10	*(13.7%)*	8	*(22.2%)*	2	*(5.4%)*
Informal employment/not regulated^e^	4	*(5.5%)*	4	*(11.1%)*	0	*(0.0%)*
Unemployed or housewives	58	*(79.5%)*	23	*(63.9%)*	35	*(94.6%)*
Retired	1	*(1.4%)*	1	*(2.8%)*	0	*(0.0%)*
**Marital status**						
Single	4	*(5.5%)*	4	*(11.1%)*	0	*(0.0%)*
Married	60	*(82.2%)*	26	*(72.2%)*	34	*(91.9%)*
Separated or divorced	7	*(9.6%)*	5	*(13.9%)*	2	*(5.4%)*
Widowed	2	*(2.7%)*	1	*(2.8%)*	1	*(2.7%)*
**Children**						
Yes	64	*(87.7%)*	28	*(77.8%)*	36	*(97.3%)*
**Number of children**^f^						
1	6	*(9.4%)*	5	*(17.9%)*	1	*(2.8%)*
2	13	*(20.3%)*	6	*(21.4%)*	7	*(19.4%)*
3	20	*(31.3%)*	10	*(35.7%)*	10	*(27.8%)*
4 or more	25	*(39.1%)*	7	*(25.0%)*	18	*(50.0%)*
**Time since migration to Spain**						
< 2 years	7	*(9.6%)*	2	*(5.6%)*	5	*(13.5%)*
2-5 years	11	*(15.1%)*	8	*(22.2%)*	3	*(8.1%)*
6-10 years	19	*(26.0%)*	4	*(11.1%)*	15	*(40.5%)*
> 10 years	36	*(49.3%)*	22	*(61.1%)*	14	*(37.8%)*
**Reason of migration**						
Economic	1	*(1.4%)*	1	*(2.8%)*	0	*(0.0%)*
Family reunification	62	*(84.9%)*	27	*(75.0%)*	35	*(94.6%)*
Tourist/student visa	7	*(8.2%)*	6	*(16.7%)*	1	*(2.7%)*
Not reported	3	*(2.7%)*	2	*(5.6%)*	1	*(2.7%)*
**Languages most spoken at home**^g^						
Spanish or Catalan^h^	14	*(19.2%)*	12	*(33.3%)*	2	*(5.4%)*
Arabic (Darija)	31	*(42.5%)*	31	*(86.1%)*	0	*(0.0%)*
Urdu	33	*(45.2%)*	0	*(0.0%)*	33	*(89.2%)*
English	5	*(6.8%)*	1	*(2.8%)*	4	*(10.8%)*
French	3	*(4.1%)*	3	*(8.3%)*	0	*(0.0%)*
Other^i^	8	*(11.0%)*	3	*(8.3%)*	5	*(13.5%)*
**Spanish language skills**						
I always need a translator	20	*(27.4%)*	8	*(22.2%)*	12	*(32.4%)*
Most of the times I need a translator	8	*(11.0%)*	3	*(8.3%)*	5	*(13.5%)*
Sometimes I need a translator	11	*(15.1%)*	6	*(16.7%)*	5	*(13.5%)*
I do not need translator at all	27	*(37.0%)*	17	*(47.2%)*	10	*(27.0%)*
**Annual household income**						
< € 12,000	11	*(15.1%)*	6	*(16.7%)*	5	*(13.5%)*
€ 12,001 - € 20,000	8	*(11.0%)*	4	*(11.1%)*	4	*(10.8%)*
> € 20,001	0	*(0.0%)*	0	*(0.0%)*	0	*(0.0%)*
I don’t know	35	*(48.0%)*	16	*(44.4%)*	19	*(51.4%)*
Prefer not to answer	14	*(19.2%)*	7	*(19.4%)*	7	*(18.9%)*
**Self-perception of religiosity**						
Very religious	23	*(31.5%)*	13	*(36.1%)*	10	*(27.0%)*
Somehow religious	43	*(58.9%)*	17	*(47.2%)*	26	*(70.3%)*
Very little religious	2	*(2.7%)*	2	*(5.6%)*	0	*(0.0%)*
Prefer not to answer	3	*(4.1%)*	2	*(5.6%)*	1	*(2.7%)*
**Muslim self-identification**						
Yes	71	*(97.3%)*	35	*(97.2%)*	36	*(97.3%)*
Prefer not to answer	1	*(1.4%)*	0	*(0.0%)*	1	*(2.7%)*
**Public health insurance**						
Yes	68	*(93.2%)*	34	*(94.4%)*	34	*(91.9%)*
No	2	*(2.7%)*	0	*(0.0%)*	2	*(5.4%)*
**Cervical cancer screening status**						
I don’t know what CC screening is	4	*(19.2%)*	0	*(0.0%)*	4	*(10.8%)*
Never screened	14	*(19.2%)*	7	*(19.4%)*	7	*(18.9%)*
Ever screened	53	*(72.6%)*	27	*(75.0%)*	26	*(70.3%)*
**Time since last cervical cancer screening**						
< 1 year	14	*(19.2%)*	8	*(22.2%)*	6	*(16.2%)*
1-3 years	21	*(28.8%)*	12	*(33.3%)*	9	*(24.3%)*
3-5 years	9	*(12.3%)*	2	*(5.6%)*	7	*(18.9%)*
> 5 years	7	*(9.6%)*	3	*(8.3%)*	4	*(10.8%)*
Unknown	2	*(2.7%)*	2	*(5.6%)*	0	*(0.0%)*

^a^ Column percentages; percentages may not add due to missing values

^b^ Percentages correspond to row percentages

^c^ Median and IQR were used as variable age was not normally distributed

^d^ Includes full-time, part time and self-employment

^e^ Includes only those reporting non regulated remunerated work, working outside their homes

^f^ Percentages were calculated among those women with children

^g^ Percentages were calculated among the total participants for each language, as multiple options could be selected in the sociodemographic questionnaire

^h^ Twelve women spoke Spanish at home. One women reported only Catalan and another reported Catalan and Spanish as languages most spoken at home

^i^ Includes Riffian (*n* = 3), Kashmiri (*n* = 1), and Punjabi (*n* = 4)

### Beliefs about health and illness

We explored participants’ perspectives on health and illness, including their beliefs regarding life and death, as well as their views on the causes, diagnosis, treatment and cure of a disease, considering both spiritual and biomedical perspectives.

#### God’s will and destiny

Both Moroccan and Pakistani women share the Islamic view that life and death are granted by God and that individuals’ fate is predetermined by God. For instance, women had the belief that being blessed with children is solely by God’s will regardless of the use of modern family planning methods, such as contraceptive pills or intrauterine devices, among others, as well as getting treated for fertility issues. The following excerpts illustrate this belief:

*“God has blessed me with a child after ten years (…) I had thyroid problems, my uterus was closed and it was down side and my eggs were not creating. They slowly started my treatment for two years and now by the grace of God I’ve a baby girl who is 4 years old and a baby boy” (FGD 1, younger Pakistani woman).*

*“(…) because pregnancy is God’s will; even though I was taking the pill, I was pregnant for five months and I didn’t know” (FGD 2, older Moroccan woman).*

Similarly, both Moroccan and Pakistani respondents perceive God as the one who ultimately controls health and illness, but also who provides the means to prevent, treat and cure the disease: *“Every disease can be cured if God wants” (FGD 3, younger Pakistani woman).* Referring to illness, women stated that *“you cannot escape from what God brings you” (SSI MC10, older Moroccan woman)*. However, in both groups, women agreed that their faith does not hinder individuals from taking responsibility of their own health and from seeking appropriate care when necessary. Some respondents emphasized that God provides the medical options for healing and, therefore, it is the responsibility of individuals to utilise them:

*“If God gives you a disease, you must accept it and fight it” (SSI MC07, older Moroccan woman).*

*“Allah himself has said that you should go for treatments” (FGD 2, younger and older Pakistani women).*

*“The fact that cancer is something that God brings you, it does not mean that you don’t have to take care of yourself, because prevention is better than cure” (FGD 0, older Moroccan woman).*

*“I believe in Him (God) and ask for His help, but I also take medicines and take care of myself, but I don’t get afraid of death” (FGD 3, older Pakistani woman).*

Two Pakistani respondents mentioned that in certain cultural contexts some people view disease as a direct punishment from God for sins committed. They stated that in Pakistan there are still individuals who believe that cancer and other illnesses are God’s punishment, implying that disease prevention can be achieved by avoiding sinful behaviours. Women of Moroccan origin did not provide any comment regarding this perspective. In fact, two of the Moroccan respondents did not make any mention to God throughout the individual interviews.

#### Spiritual prayers for healing and protection

Spiritual prayers for healing and protection continue to be practiced in Morocco and Pakistan. In both groups, some Moroccan and Pakistani women believed that engaging in ritual prayers and readings of the Qur’an may have health benefits. Several of the respondents reported to make use of these spiritual rituals as complementary sources of healing to conventional medicine, as this Pakistani woman with an educational background in Economics explained:

*“People are now less scared (of cancer) because there is chemotherapy, but there is also a verse (in the Qur’an) to listen; it is called Surah Al Rahman; it is said that if we listen this verse three times a day, it helps to cure cancer (…) I heard that in England they put it for all Muslim and non-Muslim patients and they are recovering with these verses” (SSI PC03, older Pakistani woman).*

Two respondents from Morocco also reported using spiritual treatments, such as *Ruqyah*, which consists of recitation of certain Quranic verses for healing, pain relief or protection against ‘evil eye’. Our findings also show the use of ‘transnational’ healers among Pakistani women. One participant explained that women not only consult (via phone) their families in Pakistan for advice on home remedies, but also to seek spiritual services from traditional healers in their home country.

While the mention of spiritual prayers was limited to a small number of participants, overall women believed in God’s power to protect from and cure diseases. However, they emphasized the importance of complementing faith with appropriate medical options or vice versa: “*My mum tells me to pray; she says that you can cure yourself (by praying), but she also tells me to go to the doctor and take care of myself” (SSI MC11, younger Moroccan woman).*

### Self-care attitudes and practices

Throughout the FGDs and SSIs, women shared and discussed their self-care attitudes and practices, highlighting the significance they place in their own health. This included their understanding and awareness of self-care along with the actions they take to maintain their health and well-being independently of the health system.

#### Self-care and women’s caregiver role

The prevailing sentiment among the respondents was that Moroccan and Pakistani women tend to neglect their own self-care. Instead, their priority and responsibility, or even “mission” according to one Pakistani woman, relies on taking care of their children, husbands and families. Their own well-being often takes a backseat with little importance given to their personal needs and self-care:

*“We Pakistanis don’t take care ourselves seriously, we take time out for the kids, but not for ourselves. We prepare meals for our husbands as well, but for ourselves, we don’t. We sacrifice ourselves” (FGD 3, younger Pakistani woman).*

*“A woman forgets about herself… she gets married and keeps busy with childcare, she forgets about herself, she is always the last thing” (FGD 1, older Moroccan women).*

Similarly, when the women discussed motivations and benefits of self-care, they primarily focused on the notion of taking care of themselves to ensure their ability to fulfil their responsibilities towards their families and homes, rather than focusing on their personal benefit:

*“I think (Pakistani) women should think about their health, as you are the main person (in the household) and things function through yourself. If you are healthy, then you can take care of them (husband and children) properly” (FGD 2, younger Pakistani woman).*

*“He (husband) always tells me that I need to take care of myself for them, that my children need me and that’s why I should take care of myself” (SSI PC03, older Pakistani woman).*

#### Healthy lifestyle practices

The concept of self-care is often associated with adopting healthy lifestyles, including engaging in physical activity and maintaining a healthy diet. The study participants showed little or no engagement with formal physical activities (e.g., jogging, fitness or swimming classes). Women described a discrepancy between their understanding of physical activity which was closely associated with daily living tasks and the concept of formal physical activity to maintain health, as advocated by health providers:

*“Doctors ask us ‘to drink water, eat salad and walk’. But what I do all day is walk as I’ve two grandchildren at home, so I’ve to go for the grocery, take them out. I don’t sit at home. I don’t walk like jogging but…” (FGD 3, younger Pakistani woman).*

*“Doctor said that I have to do exercise, you need to do sport for everything… I needed a solution for my health problem, not doing sport. I do ‘exercise’ at home (referring to house chores), I don’t need sport” (FGD 2, younger Moroccan woman).*

On the other hand, healthy eating was important for all participants. Both Moroccan and Pakistani women associated healthy eating with the consumption of homemade rather than processed food. However, it should be noted that this preference was partially influenced by the high prices of food:

*“I do try to eat healthy. I try not to buy packed food, those high sugar foods and, instead, I try to buy natural food, such as vegetables, fruit and fish… I try not to bring home ‘bad food’ so they don’t get used to it” (SSI MC03, younger Moroccan woman).*

“*(…) nowadays we don’t know what we eat (…) and if you are going to buy good food it is very expensive, so it is difficult…” (SSI MC01, older Moroccan woman).*

#### Religion as a self-care practice

In addition to the discussion around healthy lifestyles, when discussing the potential causes of Fatima’s CC, some participants in both groups emphasized that Muslim women do not smoke or consume alcohol. Consequently, they believed these could not be risk factors for developing cancer disease within their communities: *“We (Muslim women) don’t smoke or drink alcohol, so it can’t be because of that” (FGD 3, younger Moroccan woman*).

Some women mentioned that religious practices can serve as a form of protection against disease, *“religion cares for your health” (SSI PC06, younger Pakistani woman).* For example, most women in both groups emphasized the value of virginity and marriage not only from a religious standpoint, but also as a self-care practice. However, this belief led to a lower risk perception of acquiring sexually transmitted infections (STIs), such as Human Papillomavirus (HPV) and reduced their awareness, as this Moroccan woman expressed: *“We (Moroccan women) don’t do as many check-ups as Europeans, because in general Europeans have many sexual relationships and they get this disease (HPV infection) more than us. We only have one relationship with our husband, so this doesn’t encourage us to go for check-ups” (FGD 0, younger Moroccan woman).*

#### Self-medication

Once Pakistani women perceived themselves to be ill, they tended to self-medicate: *“We avoid going to the doctor and if we have any infection we take antibiotics and, if we feel pain, we take painkillers at home. We don’t visit them on time” (FGD 0, younger Pakistani woman)*. In contrast, a Moroccan woman referred to self-medication as an unhealthy habit: *“I avoid taking medicines as it is said they damage our kidneys and liver, so I only take what the doctor prescribes me” (SSI MC07, older Moroccan woman).*

While many women from both countries had knowledge of herbal remedies, their use was not widespread. Participants combined traditional and Western medicine, and some even expressed scepticism regarding the effectiveness of herbal remedies to treat and cure disease:

*“I don’t believe much in traditional medicine (…) It’s said that lavender with salt cures infections, I don’t know… In Morocco, people are used to healing with natural medicine, home remedies and this kind of things, and that’s because health care is expensive, medicines are expensive, so when they come to Spain they are already used to using natural remedies” (SSI MC09, younger Moroccan woman).*

### Knowledge and interest in disease prevention

We also explored Moroccan and Pakistani participants’ understanding and awareness of disease prevention. We asked them about their health-seeking behaviours and practices within the health system to prevent disease, including regular check-ups, as well as their views regarding the relevance and usefulness of early detection tests.

#### Low awareness of disease prevention

Participants perceived health as the absence of symptoms, leading them to delay seeking medical care until the appearance of discomfort or symptoms such as severe pain, as this Pakistani woman illustrated: *“My mum always said ‘we have to take care of ourselves when there is a reason; before, you don’t have to worry’” (SSI PC03, older Pakistani woman).* For example, when asked why Fatima, the fictional immigrant woman diagnosed with CC, did not see a gynaecologist for 8 years, many women in both groups answered that it was due to the lack of symptoms:

*“We Moroccan women don’t go to the doctor until we feel pain; we don’t know that cancer disease can be silent” (SSI MC11, younger Moroccan woman).*

Both Moroccan and Pakistani women reiterated that they tend to overlook their bodily concerns, leading to delays in seeking care and potentially receive a more severe diagnosis:

*“We don’t show much concern about what’s happening with our bodies and, as a result, germs in our bodies keep growing and it’s late by the time we come to know about that (cancer)” (FGD 0, younger Pakistani woman).*

Both groups of women agreed that there was a lack of interest in regular check-ups, possibly explained by the fact that regular check-ups are not commonly practiced in their countries of origin as they are in Europe:

*“We don’t have the habit of doing check-ups (…) we don’t give importance to our health… until we get ill” (FGD 2, younger Moroccan woman).*

One possible explanation for the absence of this self-care habit may be the lack of a public health system in their countries of origin, as one Pakistani woman noted: *“because there is not a public health system (in Pakistan) and people don’t have much money and the check-ups and tests are very expensive” (SSI PC07, younger Pakistani woman)*. However, in the context of the Spanish health system where preventive care services are free of charge, another Pakistani participant suggested that some women may be unaware that these services exist and are free of charge:

*“We don’t have enough information, many people don’t know that these tests exist. Even living here (Spain) we don’t know” (FGD 3, younger Pakistani woman).*

Another potential reason that emerged during the FGD with Moroccan women was the fear of being diagnosed with a disease. In some cases, these women had personal experiences of cancer within their families and had witnessed the traumatic impact of cancer, which deterred them from attending regular check-ups:

*“You don’t want to hear that you have cancer, it’s scary. So you tell yourself ‘better to leave it in God’s hands’” (FGD 0, older Moroccan woman).*

In contrast, Pakistani women did not mention this fear, although some associated regular check-ups with children’s and elderly’s health:

*“Yes, they are useful to detect something, but at my age, I’m 39… when we are older we have more health problems, that’s when we visit the doctor and we do more check-ups” (SSI PC07, younger Pakistani woman).*

#### Acceptance of screening for early detection

Although women showed limited awareness regarding the existence of asymptomatic diseases, the majority were familiar with the concept of early detection. There were differences between the two groups of respondents in their perception of the benefits of regular check-ups, particularly in relation to CC screening. Pakistani women felt more positive about the utility of preventive care services while in all FGD with Moroccan women scepticism was expressed. In this sense, some participants expressed their concern about the time interval between screening tests, particularly in the case of HPV test, which is set at 5 years:

*“I have a Spanish colleague in the office, she did the test (Pap smear) and it was ok, and six months later the cancer came out, so I think these tests are useless, because my colleague had to do the test once a year, but she did it and, between tests, in six months the cancer came out (…) so I don’t think early detection can protect from cancer” (FGD 0, younger Moroccan woman).*

This sceptic view regarding the screening tests generated a rich discussion surrounding the importance of detecting diseases, particularly cancer, at an early stage, rather than in advanced stages. For instance, when participants were asked about what Fatima (the fictional patient diagnosed with cervical cancer) could have done to prevent the disease, many women from both Moroccan and Pakistani origin agreed that she should have undergone regular check-ups: *“If she would have done regular check-ups, then she could have known about it (cancer) at an initial stage, and her treatment could have been easier and earlier” (FGD 2, older Pakistani woman)*. Participants also referred to the benefits of early detection even when only the risk factor is identified. This fact was highlighted by one participant who tested positive for HPV: *“This is like fighting against the disease, even before it comes out. If you detect the disease in the beginning is not the same as when it is developed” (SSI MC05, older Moroccan woman).*

### Changing perceptions

Finally, we identified different motivators to encourage the engagement of Moroccan and Pakistani women with preventive care services, and by the end of both group and individual interviews, we confirmed an enhanced self-awareness among participants.

#### Increasing self-awareness

Some participants questioned that Moroccan and Pakistani women do not prioritize self-care and their own well-being. Participants in the FGD advocated for being more responsible with their own health and emphasized the importance of effectively managing their time as means of promoting self-care:

*“This is not good, we should get time for ourselves as well” (FGD, Pakistani woman PG303, 36 years)*, because *“if we are not healthy, how will we do other things?” (FGD 2, older Pakistani woman).*

*“Do you work? You can get an appointment and get time to go. Children? You can get an appointment when they are at school. We must organise our own time” (FGD 2, older Moroccan woman).*

Throughout FGD and SSI, certain women felt guilty for not taking responsibility for their own health: *“I am also getting sad that I’ve not gone to the gynaecologist for the last 22 years. We should go and get the tests done” (FGD 3, older Pakistani woman).* Although most women believed that seeking care in the healthcare system was only necessary when symptoms were present and a disease needed to be treated, a growing awareness was observed at the end of the interviews in both groups regarding the possibility of feeling healthy while having undetected conditions:

*“People don’t have to wait until they are in pain to go to the doctor, they need to do check-ups from time to time, because there are diseases that have not symptoms” (SSI MC12, older Moroccan woman).*

*“… our mother-in-law was well, she was 80-something years old and she was ok, very active… but sometimes we don’t know what we have inside our bodies” (SSI PC01, younger Pakistani woman).*

#### Motivators for screening

Women seemed to be more amenable to adopt lifestyle changes when they either had a chronic condition or had a personal experience of cancer or another severe disease within their families or close friend circles.

*“The first time that we gave importance to cancer was when my sister was diagnosed with breast cancer (…) then we were more aware and we started to get screened” (SSI MC01, older Moroccan woman).*

*“Before I didn’t take care of myself at all, but now because I have problems, I have a prothesis so I cannot hold much weight (…) so I follow a diet, I eat healthy food, I drink a lot of water and I also do some exercise” (SSI PC01, younger Pakistani woman).*

Finally, both Moroccan and Pakistani women mentioned that receiving a direct invitation or request from a doctor or from the health system, such as call reminders or letters, would serve as a strong motivator for them to attend regular check-ups. One Pakistani woman even suggested the implementation of compulsory check-ups:

*“They need to call me or send me a letter to my home and remind me that I need to take an appointment. Then, I would go (for check-ups), but if it has to be from my own initiative, I just stayed telling myself ‘I’ll go, I’ll go;” (SSI MC03, younger Moroccan woman).*

*“I think rather than an invitation, it should be an obligation if it is a really important test (…) We (Pakistani) are like this, until we are not obligated, we don’t go (…) But if they obligate us, like with the COVID, then…” (SSI PC03, older Pakistani woman).*

## Discussion

As part of a broader research project to implement the new population-based CC screening programme in Catalonia, Spain, we conducted a study to enhance CC screening uptake among migrant populations. We explored concepts of self-care and prevention from the perspective of Moroccan and Pakistani immigrant women living in Barcelona province. Our research unveiled important factors to be considered to achieve successful implementation of CC screening in these study populations. Firstly, most of the study participants associated health with the merely absence of symptoms, leading them to only seek care for treatment when experiencing symptoms, rather than to prevent a disease. Secondly, they reported not having the habit of doing regular check-ups unlike European women and their self-care and prevention attitudes and practices seemed to be conditioned by cultural values. Lastly, they emphasized the need of an effective communication system to enhance their engagement and connection with preventive health services, specifically CC screening.

In our study, women represented health as ‘feeling healthy’ or not having symptoms. Therefore, the concept of self-care and prevention held little relevance for the majority, who reported taking care of themselves only when feeling unwell. This finding aligns with other studies on cancer prevention among different migrant populations which found that women did not prioritise screening when feeling healthy [4, 10, 37, 56]. Participants from both ethnic groups indicated that women in their home countries do not relate to the concept of self-care as women do in Western countries nor have the habit of undergoing regular check-ups. This observation is consistent with other studies conducted in Spain with Pakistani women [38] and with studies performed in Sweden encompassing diverse groups of immigrant women, including individuals from North Africa and Asia [22, 45]. The lack of self-care as a habit may be due to the cost of medical care in migrants’ home countries. Additionally, their lack of interest in self-care and prevention, including screening, could be influenced by their cultural understanding of these health concepts and their health practices. For example, a study conducted in Australia with Chinese migrants found that self-care went beyond a simple focus on healthy lifestyle and emphasized the need of maintaining harmony and balance in their lives, and going for medical check-ups was mainly for illness management rather than prevention [37]. In our study, we observed a similar pattern, where regular check-ups were sought only when women already had a chronic condition or when their perception of risk increased due to being exposed to cancer cases within their close circles. We also found that some women were sceptical regarding the benefit of screening due to the long intervals between tests and their own experiences of witnessing people getting ill despite having been screened.

In societies with traditional and patriarchal structures like Morocco and Pakistan, women play an important caregiver role that extends beyond their own families. In our study, most Moroccan and Pakistani immigrant women migrated to reunite with their husbands, some of whom continued living with their parental families in Spain. In this context, participants tended to prioritise the care for their children and families over their own care, reflecting the value of self-sacrifice as an integral aspect of their caregiver role. In this sense, during the FGD, a few respondents emphasized the importance of keeping healthy to fulfil their family responsibilities, suggesting that the individualistic concept of self-care often used in Western countries, in this case Spain, might be seen as ‘selfish’ behaviour and not being meaningful for specific populations. Previous studies have also identified this pattern of prioritising others’ needs over one’s own health or engaging in self-care to better provide care to others [37]. For instance, a study conducted with Somali refugees in the USA highlighted how illness adversely affected women’s relationships and their role in the family [12]. This shows that the concept of self-care in more collectivist societies, where community interdependence and familial ties are highly reinforced, such as in Morocco and Pakistan [25], may not align with a more individualistic concept of self-care which is often associated with healthy lifestyle activities, such as leisure time physical activity or regular health check-ups. This mismatch is evident in two studies that explored how culture and collectivistic families influence engagement in physical activity among Pakistani populations [54, 55]. In our study, only a very small number of women reported participating in any formal physical activity. Previous qualitative studies have also shown lower engagement in formal physical activity of Moroccan and Pakistani immigrant women in the Netherlands [44] and Spain [38], respectively. This lower engagement may be because in some cultures it may not be customary to engage in leisure time physical activity [9, 26], and therefore, immigrant women would be less likely to participate in physical activities that are not related to their daily tasks. In fact, as in the Nicolaou et al. [44] study, we found that two women from Morocco and Pakistan showed confusion regarding the distinction between physical activity associated with the tasks of daily life and leisure time or formal physical activity aimed to enhance health, considering the latter unnecessary. Also, in our study, Moroccan and Pakistani women primarily focused on healthy lifestyle choices related to food. They gave importance to consuming home-made food, not processed, aligned with Carrol et al. [12] study with Somali refugees.

Cultural and religious beliefs about health and illness can exert a significant influence on people’s health behaviours. We found that both Moroccan and Pakistani women believed that God ultimately controls health and illness and is the ultimate provider of healing. This finding aligns with previous research with Somali women in the USA [5] and the UK [1], as well as with Moroccan women in Belgium [2], which found these religious beliefs and how not taking them into account in the design of prevention programs may contribute to health inequities among Muslim populations [46, 47]. Rather than a barrier to engage in self-care and prevention practices, women in our study emphasized individuals’ responsibility to take care of their own health and use the means that God provides for prevention, treatment and cure. Other studies reflected a similar perspective among Moroccan women [24, 36], and Raymond et al. [50] suggested that religion might not be a significant barrier toward screening. We also identified the belief that disease can be a punishment from God for sins committed and ‘evil eye’. However, only two women mentioned that in their home countries individuals from rural areas and with lower education still hold this belief, which is considered an integral part of maintaining good health, as explained by Jan et al. [30] in a study on self-care views among Pakistani immigrant families in the USA.

The use of traditional medicine has a long history in Pakistan [3] and Morocco [14]. *S*tudies have shown that ethnic minorities continue to use their traditional medicinal knowledge after migration to Western countries [49]. However, in our study, we found that participants mostly used herbal medicines to complement biomedical medicine and a small number of women reported using spiritual treatments (e.g., religious prayers, health consultations to ‘transnational’ spiritual healers).

Finally, many studies have concluded that low socio-economic status is an influencing factor on the lack of adoption of self-care practices [48, 51] and, especially on CC screening uptake [11, 17, 42]. Although this aspect did not emerge in women’s discourses as a prominent theme, it is noteworthy that most participants lived in deprived areas. Nearly half of them (48%) reported that they were not aware of their annual household income, reflecting a lack of empowerment. Furthermore, over a quarter of them (26.1%) were in the lower income categories (< €20,000).

Our study has several potential limitations that need to be considered. Firstly, the findings reported here are part of a broader study aiming to improve cervical cancer screening uptake among Moroccan and Pakistani women, therefore general concepts of health and illness, and self-care and prevention have been simplified and tailored towards this aim. Nonetheless, they serve as a valuable groundwork for future exploration. Secondly, it is important to mention that the sample criteria and recruitment strategy resulted in a high representation of specific groups. Second-generation of immigrant women were excluded as the majority of them would not meet the age requirement for HPV-based CC screening in Catalonia, Spain, and women who were housewives were highly represented, especially in the Pakistani cohort. Thus, our results may not apply to women with formal employment who might also be more acculturated to the Catalan/Spanish society. Nevertheless, previous studies have shown that workforce participation by Pakistani women is generally low [6]. In the case of the Moroccan cohort, around 33% of the participants had formal or informal jobs reflecting also the low integration into the Spanish labour market of the first-generation of Moroccan immigrant women [13, 32]. Thus, our sample is fairly representative of these groups. Thirdly, the presence of research team members during the FGD along with the fact that the SSI were conducted by the first author and the RAs who were second-generation of migrants may have influenced women to provide a more closely opinion to a ‘Western’ viewpoint than they otherwise would have done, particularly, in regards to the influence of religion on their attitudes and behaviours concerning self-care and prevention. In anticipation of this, we addressed the religious questions after discussing other topics.

An important strength of this study is that the RAs and FGD moderators shared a common background and language (Darija and Urdu) with the participants. This commonality proved to be very helpful in building rapport and fostering an environment in which the respondents felt comfortable to openly share their views. Also, a clear strength is the sample diversity. We were able to recruit women with a wide range of age and educational levels, and with different length of residency in Spain, as well as from the capital city and three other semi-urban areas, giving us the possibility of capture diverse views and experiences. The mixture of FGD and SSI enriched the data as it allowed us to capture the interaction between women and reactions about their own self-care and prevention attitudes and practices [33], and more detailed accounts of individuals’ self-care and prevention experiences [18].

## Conclusion

This study provides evidence on how concepts of self-care and prevention, often offered from a ‘Western’ perspective, may not fit into other conceptualizations of self-care used in more collectivist societies and that global self-care intervention guidelines may not be meaningful or motivating for people who have different understandings of health, illness, self-care and prevention. Moreover, it emphasises the importance of understanding prevailing cultural and religious values and beliefs in relation to health, self-care and prevention, and how these evolve over time along with the exposure to new experiences and ideas. This needs to be incorporated into health promotion interventions and paves the way for future research on the potential role of religious leaders, as well as women’s social networks -including second-generation of immigrant women- as value human resources to drive the adoption of self-care practices. Therefore, to be successful in improving the coverage of CC screening programs and any other self-care intervention among underserved groups will require more culturally appropriate strategies aligned with communities’ own perceptions and priorities. By embracing these approaches, healthcare systems can truly make meaningful progress in promoting equitable access and participation in such important preventive health initiatives.

## Data Availability

The FGD and SSI guides and the codebook developed for analysis during the current study is available from the corresponding author upon reasonable request.
